# Pre-exenterative chemotherapy, a novel therapeutic approach for patients with persistent or recurrent cervical cancer

**DOI:** 10.1186/1471-2407-5-118

**Published:** 2005-09-19

**Authors:** Carlos Lopez-Graniel, Rigoberto Dolores, Lucely Cetina, Aaron Gonzalez, David Cantu, Jose Chanona, Jesus Uribe, Myrna Candelaria, Rocio Brom, Jaime de la Garza, Alfonso Duenas-Gonzalez

**Affiliations:** 1 Division of Surgery, Instituto Nacional de Cancerología, Mexico; 2 Division of Clinical Research, Instituto Nacional de Cancerología, Mexico; 3 Department of Pathology, Instituto Nacional de Cancerología, Mexico; 4 Department of CT scan, Instituto Nacional de Cancerología, Mexico; 5 Unidad de Investigación Biomédica en Cáncer. Instituto Nacional de Cancerología/Instituto de Investigaciones Biomédicas, UNAM, Mexico

## Abstract

**Background:**

Most cervical cancer patients with pelvic recurrent or persistent disease are not candidates for exenteration, therefore, they only receive palliative chemotherapy. Here we report the results of a novel treatment modality for these patients pre-exenterative chemotherapy- under the rational that the shrinking of the pelvic tumor would allow its resection.

**Methods:**

Patients with recurrent or persistent disease and no evidence of systemic disease, considered not be candidates for pelvic exenteration because of the extent of pelvic tumor, received 3-courses of platinum-based chemotherapy. Response was evaluated by CT scan and bimanual pelvic examination; however the decision to perform exenteration relied on the physical findings. Toxicity to chemotherapy was evaluated with standard criteria. Survival was analyzed with the Kaplan-Meier method.

**Results:**

Seventeen patients were studied. The median number of chemotherapy courses was 4. There were 9 patients who responded to chemotherapy, evaluated by bimanual examination and underwent pelvic exenteration. Four of them had pathological complete response. Eight patients did not respond and were not subjected to surgery. One patient died due to exenteration complications. At a median follow-up of 11 months, the median survival for the whole group was 11 months, 3 months in the non-operated and 32 months in those subjected to exenteration.

**Conclusion:**

Pre-exenterative chemotherapy is an alternative for cervical cancer patients that are no candidates for exenteration because of the extent of the pelvic disease. Its place in the management of recurrent disease needs to be investigated in randomized studies, however, its value for offering long-term survival in some of these patients with no other option than palliative care must be stressed.

## Background

Cervical cancer continues to be an important health burden with a yearly incidence of almost half a million new cases in the world and a mortality rate of about 50% [1]. Currently, locally advanced disease is treated with concurrent cisplatin-based chemoradiation [2]. However, approximately in 25% of all patients treated for cervical carcinoma, the tumor will progress or recur locally [3,4], being the most common site of recurrence the pelvis. Thus, local relapse continues to be a significant problem for these patients, as tumor persistence or local recurrence in an irradiated pelvis indicates a very dismal prognosis [5,6].

Recurrent disease can be treated by a) *chemoradiation *if the primary disease was approached with surgery; b) *palliative chemotherapy *if recurrence is considered unresectable and the primary disease was treated with radiation or chemoradiation; and c) *pelvic exenteration *for selected cases with small, central disease even if primarily treated with chemoradiation or radiation. Pelvic exenteration involves *en bloc *resection of bladder, genital tract, and rectum; it was first described by Brunschwig in 1948 [7]. This procedure has curative potential in almost half of patients undergoing this procedure [6] and it is commonly reserved for only the small subgroup of recurrent disease patients who meet the "standard" criteria for exenteration (small, central tumors). However, most pelvic recurrences do show a diffuse growth pattern fixed to one or both pelvic side walls. These fixed recurrences are felt at physical examination as "pelvic fibrosis" with or without a dominant mass. Thus, pelvic fibrosis, is an ominous finding significantly related to nodal disease and fixation to pelvic side wall [8].

Due to these facts, the vast majority of recurrent cervical cancer patients are left with no curative options, therefore it is important to search for other therapeutic alternatives in patients that are not "standardly" considered for the classical exenterative procedure. The introduction of high-dose-rate intraoperative radiation therapy (HDR-IORT) combined with radical surgical resection has widened the scope of patients who may be offered surgery [9], however, this form of radiation delivery is not widely available. In addition, despite this modality of treatment provides a reasonable local control rate in patients who have failed prior surgery and/or definitive radiation, only those with complete gross resection at completion of surgery appear to benefit from this radical approach in the salvage setting [10].

Hokel et al., have recently described the laterally extended endopelvic resection (LEER) as a novel surgical salvage therapy to a selected subset of patients with locally advanced and recurrent cervical carcinoma involving the pelvic side wall. This consists in extending the lateral resection plane of pelvic exenteration to the medial aspects of the lumbosacral plexus, sacrospinous ligament, acetabulum, and obturator membrane to allow for resection with disease-free margins [11]. With this salvage approach, they have reported a 5-year survival probability of 46% for those patients considered only for palliation with current treatment options. Although these results are highly encouraging, severe postoperative complications occur in almost half of patients and the procedure is limited to tumors sized <5 cm with a recurrence-free interval from primary radiation treatment of >5 months, and to recurrences that do not involve the larger sciatic foramen; all forms of parietal pelvic side wall disease are not suited for this procedure [12].

Currently, a combination of cisplatin and paclitaxel has shown better response rate and progression- free survival than single agent cisplatin hence, combination chemotherapy as been regarded as the standard of care in patients to be treated with systemic palliative chemotherapy [13]. Chemotherapy however, as a definitive treatment for recurrent cervical cancer has solely a palliative role, with responses that are at best partial and of short duration, as a consequence, almost all patients eventually show progression and die from their disease. Because objective responses are seen in almost a third of these patients, we reasoned that a "local" consolidation would potentially render some of these responding patients free of disease. These observations prompted us to evaluate in a pilot study, a treatment modality we have called "pre-exenterative chemotherapy" in patients with "fixed" pelvic recurrence in the aimed to shrinking the pelvic recurrent tumor to then attempt, then, a "standard" pelvic exenteration.

## Methods

All patients had histologically proven persistent or recurrent cervical carcinoma to primary radiation or chemoradiation. At the pelvic examination -under no anesthesia-, these patients were felt to have pelvic fibrosis and diagnosed as having a recurrence of diffuse infiltrative growth pattern (with or without a dominant mass) fixed or not to one or both pelvic side walls. Consequently, these patients were considered by the gynecologist team of our Institution (C L-G, A G-E, GM) to be unsuitable for pelvic exenteration regardless of the CT scan findings. Patients also had to meet the following inclusion criteria: Aged between 18 and 70 years; ECOG performance 0–1; adequate hematological, hepatic and renal functions as determined by: hemoglobin equal or higher than 10 g/L, leukocyte count higher than 4000/mm^3^, and a platelet count of at least 100 000/mm^3^, total bilirubin less than 1.5 times the normal upper limit (NUL), transaminases less than 1.5 times NUL, and normal levels of creatinine in serum; a normal posteroanterior chest X-ray as well as having the correspondent informed consent. The exclusion criteria included: severe systemic or uncontrolled disease (infection, central nervous system, metabolic, etc) that precluded the use of chemotherapy and further exenteration; concomitant treatment with any other experimental drug; mental illness and previous or concomitant malignancies except non-melanoma skin cancer. The study was approved by the Institutional Regulatory Boards.

### Pre-exenterative chemotherapy

Chemotherapy was administered in an outpatient setting. Diverse chemotherapy schedules based on cisplatin or carboplatin were used as follows: Carboplatin AUC 5, d1, paclitaxel 135 mg/m^2^, d1 and gemcitabine 800 mg/m^2 ^d1&8 (2 patients); carboplatin AUC 5–6, d1, and paclitaxel 135 mg/m^2^, d1 (3 patients); carboplatin AUC 5–6, d1, and 5FU 1 gr/m^2 ^d1-5, (3 patients); cisplatin 100 mg/m^2 ^d1, and 5FU 1 g/m^2 ^d1-5, (4 patients); and cisplatin 100 mg/m^2 ^d1 and gemcitabine 1 g/m^2 ^d1&d8 (5 patients). Courses were administered every three weeks for a maximum of 6 courses. Conventional antiemetic therapy and ancillary medications were used during drug treatment. Chemotherapy was stopped in cases of disease progression or prohibitive toxicity.

### Response and toxicity to pre-exenterative chemotherapy

Objective evaluation of response to chemotherapy using standard response criteria was not the primary objective of this study as this would have required that all patients had a well-defined and measurable mass. Instead a response to chemotherapy was defined when the pelvic disease was felt less fixed and/or the "fibrosis" was felt softer by the same team of gynecologists that performed the pre-chemotherapy evaluation. Toxicity to chemotherapy was evaluated according to the NCI Common Toxicity Criteria.

### Pelvic exenteration

After pre-exenterative chemotherapy, patients were evaluated by the same team of gynecologists through pelvic examination (CT scan was not mandatory). However, the decision to proceed or not with the surgical procedure relied only on pelvic examination and was based on whether there was or not response as evaluated with above described criteria. The other criterion for no exenteration was a worsening of the general clinical condition of the patient regardless of the pelvic examination. Patients were followed every three months after completion of all treatment.

### Survival

Overall survival was evaluated using the Kaplan-Meier method and was considered from the date of the diagnosis of the persistent or recurrent disease until the date of death of last visit.

## Results

From May 1999 to March 2003, 17 patients were studied in this pilot trial. Baseline characteristics of patients (at diagnosis of their primary disease) are shown in Table 1. The mean age of patients was 43.3 years and all, but two, were squamous histology. FIGO stage distribution was as follows: one patient was IB1, four were stage IB2, five IIB, and seven, stage IIIB. Nine received radiation alone as the definitive treatment of their primary disease, four were treated with radiation plus extrafacial complementary hysterectomy, and four patients received chemoradiation with weekly cisplatin. A complete clinical response was achieved in 13 patients after the primary treatment, three had persistent disease and one progressed. All cases accrued in this study had local pelvic relapse and the median time to progression after primary treatment was 16 months (9–120) in the 13 cases that had complete response, whereas the time to progression for the persistent or progressive disease cases was 4 months (range 2–7 months).

All patients had histological confirmation of their recurrent disease. The clinical status at entering the study is shown in Table 2. All patients complained of pelvic pain. At physical pelvic exam the disease was felt as fixed to the pelvic wall in all cases, 5 (29%) unilaterally and 12 (71%) bilaterally. This was accompanied by unilateral leg edema in six cases, hydronephrosis in three (18%) and both findings: edema and hydronephrosis in three cases (18%).

**Figure 1:**
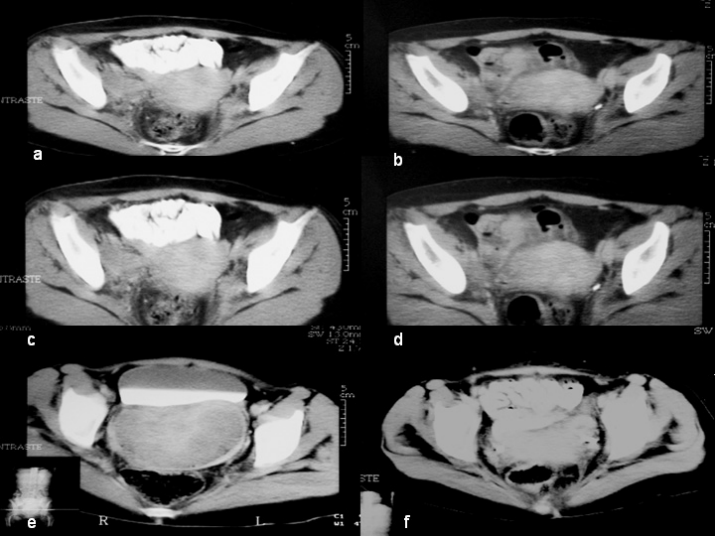
Patients with complete pathological response post-chemotherapy. CT scans of 3 patients showing residual pelvic mass after chemotherapy. Images a,c,e show CT scans pre-treatment, and images b,d,f are the post-treatment control studies. Notably, a patient (images e-f) shows a residual post-chemotherapy pelvic mass measuring 9 × 5 cm.

Table 3 depicts the overall treatment received by the patients. The median number of cycles delivered was four (range 2–6 cycles). Evaluation of response following the aforementioned subjective criteria, performed by bimanual pelvic examination, was achieved in nine patients and these underwent the exenterative procedure. Among the eight patients not exenterated, three showed progression alone, one had clinical deterioration with no change at pelvic examination and four had progression and clinical deterioration. Objective response was also evaluated using classical criteria in measurable disease (complete, no evidence of disease, partial, >50 reduction in the product of the two longest perpendicular diameters of the measurable lesion; no change or stable, <50% decrease or <25% increase, and progressive disease >25% increase). According to this, within the 8 non-operated patients, only four had pre and post-chemotherapy CT scans, three had no response and one had progression. These data correlated well with that registered in the physical examination. On the contrary, in the nine operated patients, five patients had pre and post-chemotherapy evaluation, and all five had partial response. This, also correlates with that perceived in the clinical examination. It is remarkable that within the operable patients, in no case an objective complete response was observed despite four cases had a pathological complete response. Figure 1 shows that in the three cases with pathological complete response that had pre and post chemotherapy CT scan there was residual tumor after chemotherapy.

Chemotherapy was well tolerated. The most common side effect were nausea/vomiting grade 1 and 2, mild to moderate anemia was present in half of patients; all patients presented leukopenia and neutropenia which were grade 3 in five and three patients respectively. There were no episodes of infection or bleeding (Table 4).

In regard to the exenterative procedure, a total infraelevator exenteration was done in eight cases and one had anterior supraelevator exenteration. This patient was the one with positive surgical margins in the vaginal border. It is worth mentioning that, in this case, the transoperative frozen section of the vaginal margin was reported negative; however, the definitive histological analysis showed disease. The definitive histological analysis of the surgical specimens showed a complete pathological response in 4 cases, a residual disease ≤2 cm in four cases, and one case with a residual measuring 8 cm. In seven patients, the urinary diversion consisted of an ileocolonic conduit and an ileal conduit in two cases. Colostomy was done in the eight cases undergoing total exenteration, (Table 5). Regarding surgical morbidity, the mean surgical time was 6.3 hours (range 4.3–8); the mean of bleeding was 1860 mL (range 600–6000 mL). All patients required at least one unit of red blood cells being the mean number of units 3.4 (range 1–6). The mean hospital stay was 11.7 days (range 6–41), and the mean stay in the intensive care unit was 1.8 days (0–12 days). Among the perioperative and post-operative complications, one patient (11%) presented intestinal occlusion that resolved with non-operative measures, one had massive bleeding during the surgery (11%), there was one case with urinary fistula (11%) and two cases showed a perineal infection (22%). One patient (11%) died at day 120 post-exenteration due to sepsis. This patient was one of the four with a pathological complete response (Table 6).

**Figure 2:**
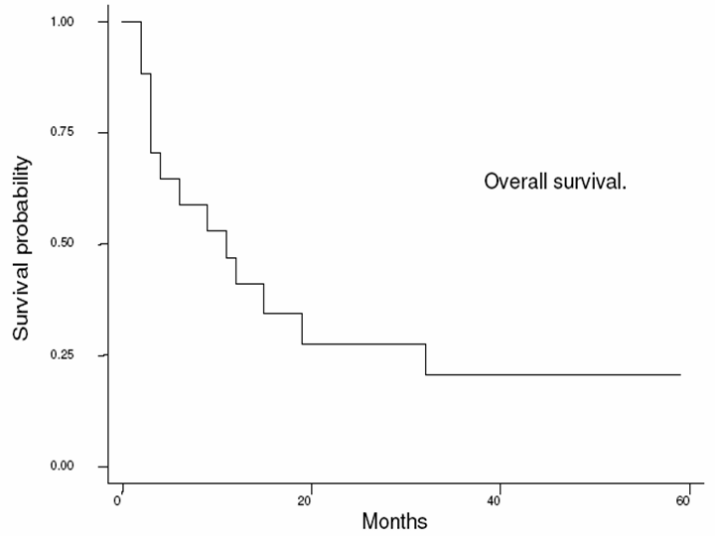
Overall survival in the intention to treat; four out of 17 are alive for a median survival of 11 months.

**Figure 3:**
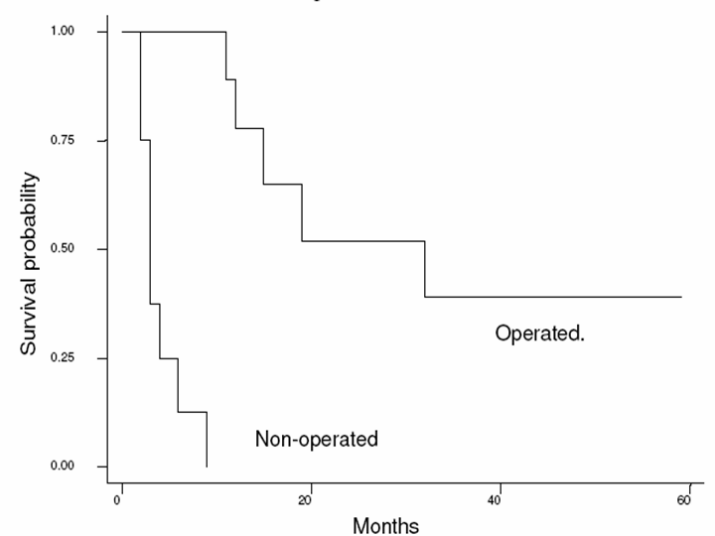
Survival in the operated and non-operated patients; median survival was 3 versus 32 months respectively. This difference is highly significant.

All patients not subjected to exenteration showed disease progression and died within the ensuing months, being the median survival of only 3 months. The status of the operated patients is as follows: patient 1: Path CR, alive without disease at 62 months, patient 2: Path CR, alive without disease 59 months; patient 3: Residual of 2 cm, local and regional recurrence at 7 months post-exenteration, patient 4: Residual of 2 cm, alive without disease 52 months, patient 5: residual of 2 cm, local recurrence at 10 months, patient 6: residual of 2 cm, local recurrence at 7 months, patient 7: Path CR died at four months from surgical complications, patient 8: Path CR, died at 20 months from liver recurrence; patient 9: Residual of 8 cm, alive without disease 13 months. Thus, four of the nine operated patients are alive without disease. Median survival in the intention to treat was 11 months, being 3 versus 32 in the non-operated versus those that underwent exenteration (Figures 2 and 3).

## Discussion

Although pelvic exenteration plays a definitive role in the management of recurrent cervical carcinoma, its impact in terms of the proportion of cervical cancer patients who benefit from such radical procedure has remained unchanged because it continues to be indicated in only very selected patients with small central pelvic recurrences. This fact, along with better medical support such as routine use of prophylactic heparin, antibiotics, nutritional support, and routine postoperative monitoring, have reduced the morbidity from pelvic exenteration [14].

In order to increase the proportion of patients in whom this salvage therapy could be attempted, we developed the modality of "pre-exenterative chemotherapy" under the rationale that systemic chemotherapy would allow the obtaining free surgical margins in patients undergoing the "standard" supra or infraelevator pelvic exenteration operation in situations where the extent of pelvic disease predicts that negative surgical margins would unlikely be obtained. The results of this pilot study demonstrate the feasibility of this approach, as nine (53%) out of the 17 patients included in this trial underwent pelvic exenteration obtaining disease-free margins in all but one case; four of them are alive without disease.

The clinical characteristics of the patients included in this study are remarkable in the sense that all of them were considered no suitable for pelvic exenteration according to standard criteria by the team of gynecologists of our Institution. This special subgroup of patients with recurrent disease is better defined if we look at their clinical characteristics: 13 of them relapsed at a median time of only 16 months whereas four were refractory to primary treatment and progressed within two to seven months All of them complained of pelvic pain, five had unilateral leg edema, three presented hydronephrosis and three cases had both signs. Both the short disease-free interval and the presence of one or more of the typical triad of signs and symptoms are either contraindications or factors predicting a very poor outcome after exenteration in most of the reported series [15-20].

Selecting the true candidates for pelvic exenteration is a difficult clinical dilemma in patients with recurrent cervical cancer after radiation therapy. Despite very thorough preoperative investigation, inoperable disease is discovered at the time of laparotomy in up to 50% of cases [21]. CT scanning is still one of the most extensively used diagnostic tool, however it may be difficult to differentiate recurrence from postoperative and post-radiation fibrosis [22,23]. MRI has been regarded superior to CT scan in visualization of the tumor and parametrial invasion in primary tumors [24]; dynamic contrast-enhanced subtraction MRI may differentiate between recurrent tumor and benign conditions [25]. However, when MRI has been used for determining surgical elegibility for pelvic exenteration its accuracy has been of 83% [26]. The difficulties encountered by the common imaging methods for evaluating the extent of disease, such as CT scan and MRI [27], have led to propose laparoscopy to select candidates to undergo the procedure [28,29], which proved to be effective as it may spare unnecessary laparotomy in half of the candidates patients [28].

It must be stressed, however, that all these imaging and laparoscopy efforts to predict resectability and avoid aborted exenterations are done in the setting of "classic indications" of pelvic exenterations, where the ultimate goals are increasing the efficacy of the procedure in terms of disease control and decreased morbidity and mortality. However, our approach in aimed at increasing the proportion of patients in whom this salvage therapy could be attempted under the rationale that systemic chemotherapy would allow obtaining free surgical margins in situations where the extent of pelvic disease predicts that negative surgical margins would unlikely be obtained and therefore exenteration could not be offered to these patients.

These considerations led us to rely on bimanual pelvic examination, which is a subjective test, as our principal criterion for deciding to perform the exenteration (as long as there was no regional or systemic disease evaluated by CT scan). We acknowledge that it would had been very valuable to have an objective pre and post-chemotherapy evaluation of the response by a CT scan, RMI, and/or PET scan in all the cases, however, only nine cases had CT scan pre and post therapy. Notwithstanding it is interesting to notice that although all our patients were "felt" by physical exam to have side wall fixation, the CT scan confirmed it only in six cases based on the criterion of having a less than 3 mm separation of the tumor from the pelvic muscles and/or vascular encasement [30]. It is important also to notice that in the four cases that were not candidates for exenteration after chemotherapy and had pre and post-chemotherapy CT scans, there was no response in three and progression in one, matching closely the findings of bimanual pelvic examinations. In contrast, the five cases that underwent exenteration and were felt to have responded by physical examination, with the criteria used, had partial responses according to the standard WHO criteria suggesting that, after all, CT scan can be a reliable method for evaluating the response to chemotherapy in the setting of pelvic recurrences in a previously irradiated site.

Nevertheless, an important observation is the fact that in three out of the four cases that achieved a pathological complete response, the CT scan was clearly positive for the presence of tumor. This is shown in figure 1, standing out patient 3 (Figure 1e–f) in whom the residual mass after chemotherapy (1e) measured 9 × 5 cm. This finding might suggest that exenteration could be useful after any degree of response to chemotherapy because the actual response to chemotherapy could be of greater magnitude than predicted by CT scan.

The use of chemotherapy in the palliative setting of persistent or recurrent pelvic disease, particularly in a patient who has received definitive radiation or chemoradiation treatment has very limited value. In a review on results of 190 advanced or recurrent disease patients treated with 14 different chemotherapy protocols, the overall response rate was 20.0% (4.2% complete response; 15.8% partial response), with a median response duration of 4.8 months [31]. In a recent phase III study comparing cisplatin versus cisplatin paclitaxel, the response rate in the subgroup with pelvic disease revealed onjective responses in 14(21%) of 66 patients treated with cisplatin alone and in 17(33%) of 52 patients treated with the combination, however, median survival was the same, 8.8 months and 9.7 months, respectively [13]. The response reported here using a platinum-based scheme was 55% (partial responses) in the nine patients with pre and post-chemotherapy evaluation by CT scan, however it was only 29% taking into account the 17 patients evaluated. This response rate as well as the observed toxicity, is within the range expected but no assumptions can be made on the efficacy of any of the schemes used.

So far there is information of the efficacy of chemotherapy in terms of pathological response in recurrent or advanced cervical cancer because chemotherapy is only used as a palliative measure and surgery is commonly not affered after chemotherapy. Here we demonstrate a pathological complete response rate of 44% in the nine patients treated (23.5% taking into the 17 patients), which is higher than obtained in neoadjuvant trials in locally advanced cervical cancer utilizing platinum-based schemes with newer drugs, such as gemcitabine [32,33], vinorelbine [34], paclitaxel [35,36], and irinotecan [37]. It is worthwhile noticing that the neoadjuvant trials with lower complete response rates were those that subjected more patients to surgery. The two studies with the lowest complete responses, 16% and 17% operated 89% and 95% of patients, respectively [35,36], whereas in the trial with 37.5% of complete response, the surgery rate was only 52% [34]. These data may explain our 44% of pathological complete response rates, since only 52.9% of our patients underwent surgery.

A noticeable finding of the present report is that the median survival of 11 months compares favorably with studies using systemic chemotherapy in the palliative setting, ranging from 6 to 10 months [38,39]; however, we must stress that half of patients (the non-operated) had a median survival of only 3 months which suggest that the patient population of patients had indeed very unfavorable clinical characteristics. Of outmost importance is the fact that from the operated patients four achieved pathological complete response despite having tomographic evidence of residual tumor and the median survival for these 9 patients taken to exenterative surgery was 32 months. The fact that there was "gross persistent" disease in the CT scan after chemotherapy support our view that the overestimation of pelvic disease, either by pelvic examination and/or imaging methods, hinder offering a potentially curative surgery to a huge proportion of patients with pelvic recurrence of cervical carcinoma. On these bases, we are just to start a randomized study comparing pre-exenterative chemotherapy and exenteration versus palliative chemotherapy alone in patients with pelvic disease that do not meet the criteria for pelvic exenteration.

## Conclusion

The therapeutic modality here reported, called pre-exenterative chemotherapy, is a therapeutic alternative for cervical cancer patients with recurrent or persistent disease limited to the pelvis not usually considered candidates for "classical" pelvic exenteration. Its value in the management of recurrent disease needs to be confirmed in a randomized phase III study.

## Competing interests

The author(s) declare that they have no competing interests.

## Authors' contributions

R D-V, J U-D, JC and RB participated in data collection and analysis; LC, DC, C L-G, A G-E and A D-G managed the patients; MC, and J de la G critically read and participated in the manuscript; additionally C L-G and A D-G conceived and wrote the manuscript.

## Pre-publication history

The pre-publication history for this paper can be accessed here:



## Figures and Tables

**Table 1:** Characteristics of patients

Number	17
Age	43.3 (29–55)
Histology	
Squamous	15
Adenocarcinoma	2
FIGO Stage at diagnosis	
1B1	1
1B2	4
IIB	5
IIIB	7
Primary Treatment	
RT alone*	9
RT alone + Adj Hyst	4
Chemoradiation**	4
Response to Primary Treatment	
Complete response	13
Persistence	3
Progression	1
Months to treatment failure	
Recurrence (13 pts)	16 (9–120)
progression (4 pts)	4 (2–7)

* Radiation: 50Gy of external radiation plus brachytherapy to achieve at least 85Gy to point A. ** Six weekly applications of cisplatin at 40 mg/m^2 ^during external radiation.

**Table 2:** Clinical status of patients at entering the study

Sign/Symptom	Number	(%)
Pelvic pain	17	100
Fixation to pelvic side wall*		
Unilateral	5	29
Bilateral	12	71
Ipsilateral leg edema	6	36
Hydronefrosis	3	18
Leg edema/hydronephrosis	3	18

* As determined by bimanual pelvic examination.

**Table 3:** Overall treatment

Median number of cycles	4
Exenterated	9
No exenterated	8
Reason for no exenteration	
Progression	3
Clinical deterioration	1
Both	4

**Table 4:** Toxicity to chemotherapy. (expressed by patient).

Toxicity		Grades	(%)		
	0	1	2	3	4

Nausea/Vomiting	0	10	6	1	0
Diarrhea	13	2	2	0	0
Neuropathy	15	1	1	0	0
Anemia	6	4	4	3	0
Leukopenia	0	3	9	5	0
Granulocytopenia	0	6	8	3	0
Thrombopenia	12	2	0	3	0

**Table 5:** Surgical data and pathological response

Exenterated	9
Total infraelevator	8
Anterior supraelevator	1
	
Pathological Response	
Complete	4
Partial	5
≤ 2 cm residual	4
8 cm residual	1
	
Surgical margins	
Negative	8
Positive	1
	
Ileocolonic conduit	7
Ileal conduit	2
Colostomy	8

**Table 6:** 

Surgical morbidity	Mean	Range
Surgical time	6.3	4.3–8
Bleeding	1860 mL	600–1600
Units transfused	3.4	1–6
Hospital stay	11-7	6–41
Intensive Care Unit stay	1.8	0–12
Complication (events)		
Intestinal occlusion	1 (11%)	
Massive bleeding	1 (11%)	
Urinary fistula	1 (11%)	
Perineal infection	2 (22%)	
Death	1 (11%)*	

The patient died (fistula and infection) at 4 months post-exenteration. This patient had pathological complete response.
